# Rapid resolution of non-segmental vitiligo in a patient treated with abrocitinib: A case report

**DOI:** 10.1177/2050313X241231527

**Published:** 2024-02-14

**Authors:** Seyyon Satkunanathan, Mina Boshra, Janis Chang, Reetesh Bose

**Affiliations:** 1 Faculty of Health Sciences, University of Ottawa, Ottawa, ON, Canada; 2 Faculty of Medicine, University of Ottawa, Ottawa, ON, Canada; 3 Division of Dermatology, Department of Medicine, University of Ottawa and The Ottawa Hospital, Ottawa, ON, Canada

**Keywords:** Vitiligo, abrocitinib, Janus Kinase inhibitors, depigmentation

## Abstract

Vitiligo is a common, autoimmune, depigmenting disorder of the skin. Janus Kinase inhibitors have emerged as promising topical and oral therapeutic options for vitiligo. There have been no reports of vitiligo being treated with oral Abrocitinib, a selective Janus Kinase 1 inhibitor approved for the treatment of moderate to severe atopic dermatitis. Here, we present a 61-year-old male with acrofacial vitiligo who had repigmentation plateau with twice daily tacrolimus 0.1% ointment, oral ginkgo biloba, and oral minipulse prednisone × 4 months; however, they had significant improvement after taking abrocitinib 100 mg per day for 2 months. He was able to transition topical tacrolimus twice weekly monotherapy for maintenance. This report shows that oral Janus Kinase inhibitors may be a useful option for the treatment of recalcitrant vitiligo. Results of ongoing randomized control trials are needed to determine the durability and safety of oral Janus Kinase inhibitors long-term.

## Introduction

Vitiligo is a chronic, acquired dermatosis characterized by the progressive autoimmune mediated destruction of melanocytes, the cells responsible for producing the skin pigment melanin. This results in depigmented, white patches, which can affect any part of the skin, mucosa and hair. The condition is estimated to affect approximately 0.1%–3% of the population worldwide,^1^ and does not have a particular predilection based on age, gender, or ethnicity, however, it can be particularly psychosocially and culturally impactful for younger patients and those with darker skin types.^2^ The convergence theory states that a complex interplay of stress, buildup of toxic compounds, infection, autoimmunity, mutations, altered cellular environment, and impaired melanocyte migration are all linked to its etiology.^3^

There is currently no cure for vitiligo, and treatment options aim to achieve three primary goals: stopping the autoimmune mediated destruction of melanocytes, influencing melanocytes to repopulate the affected area, and to influence melanocytes to produce melanin. Therapeutic approaches may include topical corticosteroids, calcineurin inhibitors, phototherapy, and surgical procedures such as skin grafting or melanocyte transplantation.^2^

The emergence of JAK inhibitors (JAKi) as potential treatment options for vitiligo has sparked interest in exploring novel avenues for managing the condition. While vitiligo treatment options have improved, challenges remain in achieving consistent, satisfactory, lasting results, especially in cases of extensive or refractory disease. Further research is essential to gain a deeper understanding of vitiligo’s underlying mechanisms and to develop more effective and personalized treatment strategies for individuals affected by this condition.^2^

## Case report

**Figure 1.**
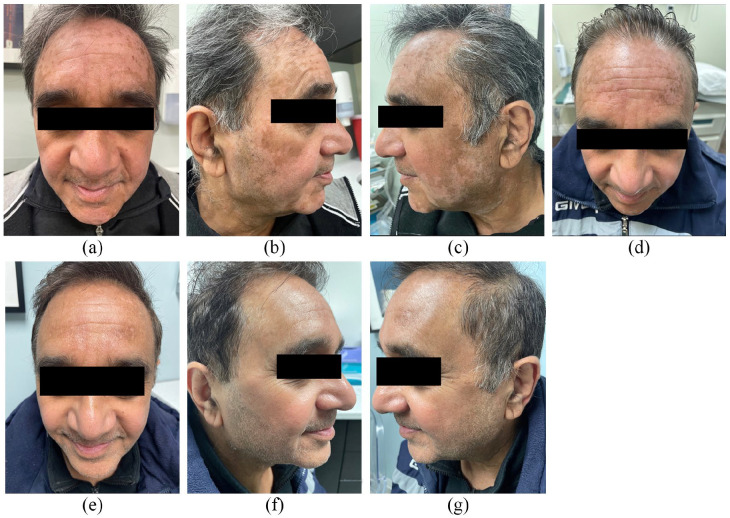
(a)–(c) Baseline (November 2022), (d) 4 months of protopic (March 2023) (e)–(g) (November 2023) after stopping abrocitinib for 4 months.

A 61-year-old male with diabetes and no personal or family history of atopy, thyroid disease or vitiligo presented with active non-segmental, acrofacial vitiligo. They had developed depigmented patches on hands and face since May 2022. He was not previously treated with any prescribed medications. He was referred to dermatology in November 2022, (Figure 1(a)) and was prescribed tacrolimus 0.1% ointment to be applied twice a day (BID). They were developing new expanding depigmented macules and patches and so were started on oral mini-pulse prednisone 10 mg two consecutive days per week. He took this for 4 months as well as ginkgo biloba 120 mg intermittently.

At 4-month follow-up (March 2023), some larger patches had partially improved, but this had plateaued by month 2, and new depigmented macules were noted on jawline and scalp. They found results unsatisfactory and were bothered by the greasy ointment, and needing to take multiple oral treatments and supplements which were causing Gastrointestinal (GI) upset and raised blood sugar.

Due to lack of access to topical JAKi and phototherapy at the time, he was started on abrocitinib 100 mg daily (April 2023). He noted significant improvement of their vitiligo at the one-month mark (May 2023). At 2-month follow up on JAKi (July 2023), he had significant repigmentation, no noted side effects, and no recurrence or progression of vitiligo patches. After 2 months on abrocitinib, patient was switched back to tacrolimus 0.1% daily for maintenance with no relapse and further repigmentation at 4 months off abrocitinib Figure 1(e)–(g).

## Discussion

Due to significant physical, psychosocial and cultural burden of vitiligo, various treatment modalities have been explored but achieving long lasting satisfactory results remains challenging.^4^ Topical corticosteroids and calcineurin inhibitors are commonly used for mild cases, but their effectiveness are limited in more extensive disease and refractory cases. Phototherapy can stimulate melanocyte activity but requires multiple weekly sessions that can span over a 6-month duration and can darken the unaffected skin. Surgical interventions including skin grafting and cell transfer techniques are proving helpful in stable and segmental types of vitiligo, but are more invasive, can only treat a limited surface area, and may have associated complications such as infection, scarring, and cobblestone type dyspigmentation. Cosmetic camouflage and psychological support help address esthetic concerns and emotional impact, respectively. While these strategies offer benefits, individual response can vary, underscoring the need for continuous research and personalized approaches to vitiligo care.^5^

JAKi have shown promise in the treatment of various autoimmune and inflammatory conditions. Their mechanism of action revolves around targeting the Janus Kinase enzymes, specifically JAK1, JAK2, JAK3, and TYK2, which play a critical role in cytokine signaling and immune response regulation.^6^ JAKi disrupt the intracellular signaling pathways responsible for the activation of immune cells and the production of pro-inflammatory cytokines that are involved in vitiligo pathogenesis. Thus, JAKi can potentially prevent further depigmentation and stimulate repigmentation by allowing the melanocytes to regenerate.^6,7^

Reported side effects of oral JAKi include erythema, transient acne, hyperpigmentation, headache, transient hyperlipidemia and creatine kinase, nasopharyngitis, infection, and weight gain.^8^ Major adverse cardiac events, thromboembolic events, malignancy and thrombocytopenia are rarer side effects, mainly seen in studies.^9^ Due to JAKi being immunosuppressants, increased risk of infection is also a concern. The rheumatoid arthritis trials show that most infections are not severe and did not usually result in treatment discontinuation. Instead, the risk of serious infections were comparable to biologics in that population.^10^ An increase in venous thromboembolism risk has been noted; however, this risk is shown to be vary depending on the condition that is being treated by JAKi.^9^

According to a recent review on JAKi in vitiligo treatment,^11^ it was found that topical application of ruxolitinib demonstrated significant skin re-pigmentation in patients with vitiligo and alopecia areata without systemic complications. Tofacitinib, a non-selective JAKi, has been successful in treating skin disorders including plaque psoriasis, atopic dermatitis, and alopecia areata, but has a less favorable side effect profile compared to more selective JAKi. Baricitinib, a JAK1 and JAK2 inhibitor, led to complete re-pigmentation in one vitiligo case.^12^ Ongoing research is investigating the combination of Baricitinib and phototherapy for vitiligo.^13^ Other JAKi such as Baricitinib, brepocitinib, cerdulatinib, Ifidancitinib, and ritlecitinib are under investigation for vitiligo, potentially shedding light on additional inflammatory pathways involved in the condition.^5^

This case report contributes to the growing evidence demonstrating the effectiveness of JAKi in treating vitiligo and shows that selective JAK1 inhibition such as with abrocitinib may be able to rapidly improve facial vitiligo. Uniquely, this case report shows maintenance of clearance and further improvement while off abrocitinib. Randomized controlled studies with larger numbers of patients are needed to characterize the true effectiveness and safety of abrocitinib and other oral JAKi for the treatment of vitiligo.
